# Diagnostic accuracy of transthoracic echocardiography to detect structural abnormalities of the outflow graft in patients with left ventricular assist devices

**DOI:** 10.1093/ehjimp/qyag017

**Published:** 2026-03-09

**Authors:** Tasuku Sato, Takeo Fujino, Kayo Misumi, Toru Hashimoto, Takamori Kakino, Akira Shiose, Kohtaro Abe

**Affiliations:** Heart Center, Kyushu University Hospital, 3-1-1, Maidashi, Higashi-ku,Fukuoka 812-8582, Japan; Department of Advanced Cardiopulmonary Failure, Faculty of Medical Sciences, Kyushu University, 3-1-1, Maidashi, Higashi-ku, Fukuoka 812-8582, Japan; Department of Cardiovascular Medicine, Faculty of Medical Sciences, Kyushu University, 3-1-1, Maidashi, Higashi-ku, Fukuoka 812-8582, Japan; Department of Cardiovascular Medicine, Faculty of Medical Sciences, Kyushu University, 3-1-1, Maidashi, Higashi-ku, Fukuoka 812-8582, Japan; Department of Cardiovascular Medicine, Faculty of Medical Sciences, Kyushu University, 3-1-1, Maidashi, Higashi-ku, Fukuoka 812-8582, Japan; Department of Cardiovascular Medicine, Faculty of Medical Sciences, Kyushu University, 3-1-1, Maidashi, Higashi-ku, Fukuoka 812-8582, Japan; Department of Cardiovascular Surgery, Faculty of Medical Sciences, Kyushu University, 3-1-1, Maidashi, Higashi-ku, Fukuoka 812-8582, Japan; Department of Cardiovascular Medicine, Faculty of Medical Sciences, Kyushu University, 3-1-1, Maidashi, Higashi-ku, Fukuoka 812-8582, Japan

**Keywords:** transthoracic echocardiography, left ventricular assist device, outflow graft, external outflow graft obstruction

## Abstract

**Aims:**

Structural abnormalities of the outflow graft (OG), such as kinking or external obstruction, are recognized as serious complications in patients with left ventricular assist devices (LVADs). Generally, these abnormalities can be evaluated by contrast-enhanced computed tomography (CT) or angiography; however, the utility of transthoracic echocardiography (TTE) remains unclear.

**Methods and results:**

This single-centre retrospective study included adult patients with LVADs who underwent both TTE and contrast-enhanced CT between January 2015 and December 2022. TTE evaluation employed a standardized protocol using subcostal and right parasternal approaches. OG structural abnormalities were defined as bending of ≥90°, or stenosis of ≥50%. The diagnostic accuracy of TTE was assessed using CT as the reference standard. Of 90 patients with LVADs, 54 patients (62 examinations of both TTE and CT) met inclusion criteria. Among 62 examinations, OG structural abnormalities were identified in 18 examinations by CT (12 proximal, 7 distal, 1 both). TTE demonstrated a sensitivity of 61% and specificity of 100% overall. Sensitivity was 33% for proximal and 71% for distal abnormalities.

**Conclusion:**

TTE is an accurate and non-invasive modality for detecting OG structural abnormalities, particularly in distal segment. However, its diagnostic performance for proximal OG lesions is limited, likely due to acoustic interference and anatomical constraints. Further refinement of imaging techniques may enhance the utility of TTE in LVAD management.

## Graphical Abstract

**Graphical Abstract:**
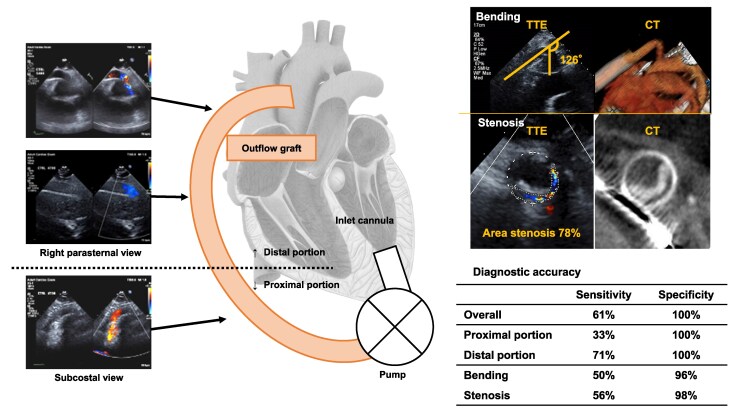

## Introduction

Left ventricular assist device (LVAD) therapy is a treatment option for patients with advanced heart failure. LVAD have demonstrated remarkable benefits for patients with advanced heart failure refractory to medical treatment, enabling them to live at home with dramatically reduced heart failure symptoms. An LVAD aspirates blood from the left ventricular apex through the inflow cannula, and delivers it to the ascending aorta via the outflow graft (OG). The OG originates from the pump located beneath the left costal arch and runs along the right side of the heart to the ascending aorta. OG structural abnormalities such as external OG obstruction (eOGO) and kinking are known complications after LVAD implantation, which has been generally reported using angiography or computed tomography (CT).^1–5^ On the other hand, few studies have evaluated the diagnostic utility of transthoracic echocardiography (TTE), a non-invasive and repeatable imaging modality, for detecting OG structural abnormalities and there is no established method for observing the entire OG by TTE. At our institution, we have standardized a method for routine evaluation of the OG from the distal to the proximal segments in order to detect morphological changes at an early stage. However, to the best of our knowledge, no prior studies have reported the diagnostic accuracy of TTE for detecting structural abnormalities of the OG. In this study, we evaluated the diagnostic accuracy of our institutional TTE-based method for assessing OG morphology.

## Methods

### Study design

This is a single-centre, retrospective and observational study. The study was approved by the Institutional Review Board (approval number 24127-00) and conducted in accordance with the principles of the Declaration of Helsinki. Informed consent was obtained through an opt-out procedure on the website.

### Study protocol

The study included adult cases who underwent LVAD implantation at our institution between January 2015 and December 2022. Both contrast-enhanced CT and TTE images evaluating the OG were retrospectively analysed. Cases were excluded if the interval between CT and TTE examinations exceeded 1 month, if our institutional protocol was not used for OG evaluation, or if both TTE and CT examinations were repeated within six months in the same case.

### Institutional protocol for scanning the OG

**Figure 1:**
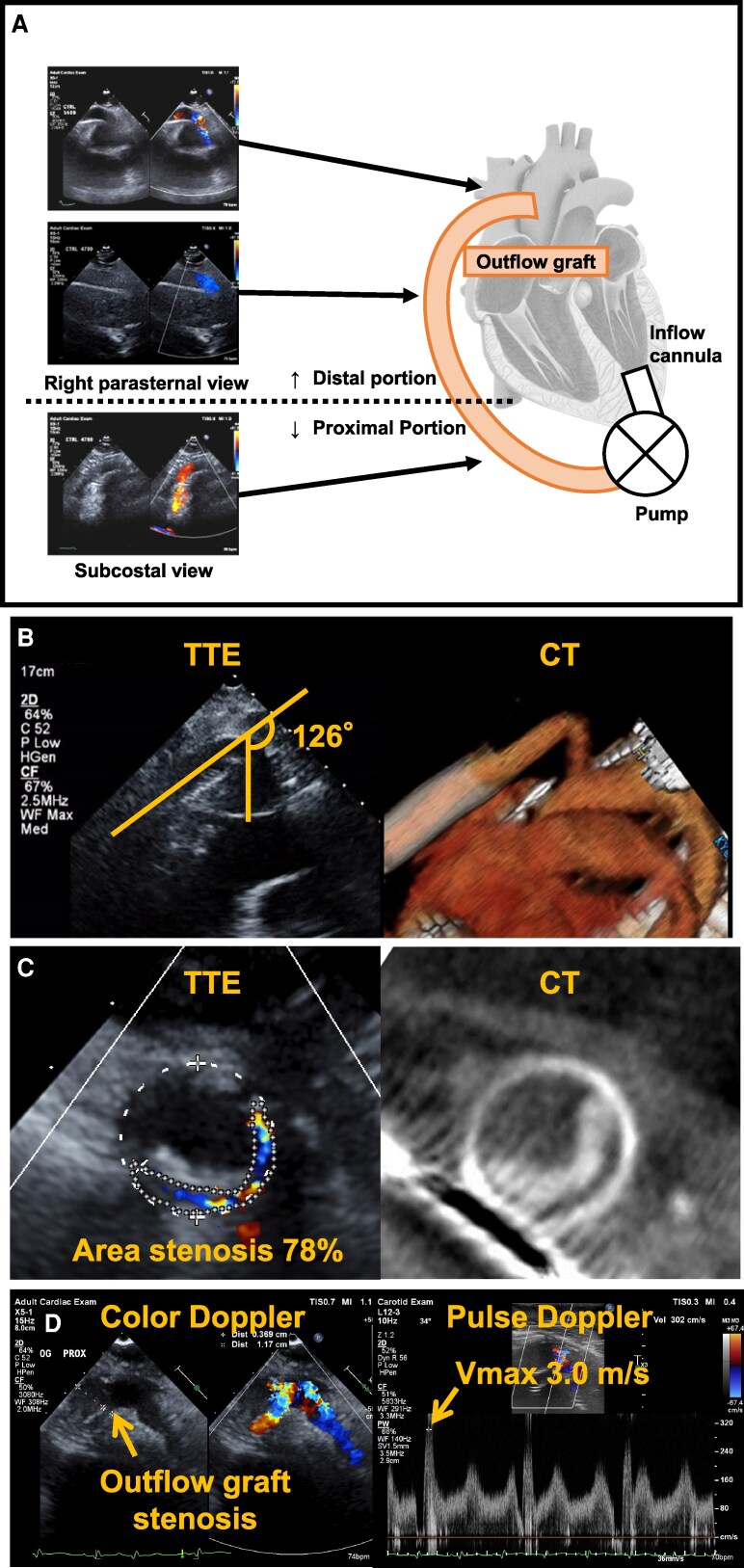
A schema showing the position of the OG and typical TTE and CT images of OG structural abnormalities. (*A*) Echocardiographic views at each OG position. (*B*) Bending at the distal portion of the OG (bending angle 126°; left, TTE; right, CT). (*C*) Stenosis due to biodebris at the proximal portion of the OG (78% area stenosis, cross-sectional view; left, TTE; right, CT). (*D*) Colour Doppler and pulsed-wave Doppler images demonstrating an increased peak flow velocity at the stenosis site (Vmax = 3.0 m/s). OG, outflow graft; TTE, transthoracic echocardiography; CT, computed tomography.

The standardized institutional protocol is described below. As shown in *Figure 1A*, we divided the structure of the OG into proximal and distal portions. The proximal portion was defined as the part inferior to the xiphoid process, and the distal portion was defined as the part superior to it. The proximal portion of the OG was evaluated using a subcostal approach, and the distal portion using a right parasternal approach.^6^ On the proximal portion, the OG running above the diaphragm was searched for using the subcostal approach with colour Doppler and pulse-wave Doppler to confirm the waveform. To evaluate the distal portion, the probe was slid towards the right sternal region with colour Doppler guidance and traced to the anastomosis to the ascending aorta. The visualized OG was described in both long-axis view and short-axis view and followed to the extent possible. To improve colour Doppler detection, the colour Doppler gain and frequency were appropriately adjusted.

### Definition of OG structural abnormalities

OG structural abnormalities were defined as a bend of ≥90°, or a lumen stenosis of ≥50% detected by CT. The angle and stenosis of the OG were measured after adjusting the imaging plane using the multiplanar reconstruction method. The same definitions of OG structural abnormalities were used for TTE diagnosis (*Figure 1B* and *C*). In addition, peak flow velocity (Vmax) was obtained using pulsed-wave and/or continuous-wave Doppler. An elevated Vmax >2.0 m/s was considered suggestive of possible graft stenosis and was used as a supportive Doppler finding of OG structural abnormalities (*Figure 1D*).

### Statistical analysis

Values are expressed as the median (interquartile range) or number (percent), as appropriate. The sensitivity and specificity of TTE were calculated based on the results of contrast-enhanced CT examinations. Ninety-five percent confidence intervals (95% CIs) were also calculated using the Clopper–Pearson method.

## Results

### Baseline characteristics

Of the 90 patients who had undergone LVAD implantation during the study period, 54 patients who underwent TTE and contrast-enhanced CT at the same time were enrolled. Among the 54 patients, eight patients had two pairs of simultaneous TTE and CT at different times, and thus 62 examinations were included and analysed.

Baseline characteristics of enrolled patients are shown in *Table 1*. The most common underlying disease was idiopathic dilated cardiomyopathy, observed in 34 patients (63%). HeartMate II (Abbott) device was used in 22 patients (41%), followed by HeartMate 3 (Abbott) in 19 patients (35%). The median post-operative period was 73 [25–936] days. The left ventricular end-diastolic dimension was 56 [46–64] mm.

**Table 1:** Baseline characteristics

Variables	Value
Demographics (*n* = 54)	
Age (years)	48 [35–55]
Male, *n* (%)	38 (70)
Body surface area (m^2^)	1.68 [1.56–1.86]
Heart failure aetiology, *n* (%)	
Dilated cardiomyopathy	34 (63)
Ischaemic heart disease	7 (13)
End-stage hypertrophic cardiomyopathy	3 (6)
Post-myocarditis	3 (6)
Congenital heart disease	2 (3)
Drug-induced cardiomyopathy	2 (3)
Others	3 (6)
LVAD device, *n* (%)	
HeartMate II	22 (41)
HeartMate 3	19 (35)
Jarvik2000	5 (9)
HVAD	5 (9)
EVAHEART2	3 (6)
Laboratory findings (*n* = 62)	
Haemoglobin (g/dL)	11.6 [10.1–13.1]
Brain natriuretic peptide (pg/mL)	117 [60–208]
Estimated glomerular filtration rate (mL/min/1.73 m^2^)	81 [58–109]
Creatinine (mg/dL)	0.75 [0.59–1.11]
Aspartate transaminase (U/L)	22 [18–34]
Alanine aminotransferase (U/L)	16 [11–22]
Lactate dehydrogenase (U/L)	282 [219–372]
C-reactive protein (mg/dL)	0.9 [0.2–4.2]
D-dimer (μg/mL)	5.6 [1.7–10.8]
Echocardiographic parameters (*n* = 62)	
Left ventricular ejection fraction (%)	20 [14–32]
Left ventricular end-diastolic diameter (mm)	56 [46–64]
Left ventricular end-systolic diameter (mm)	50 [41–61]
Aortic valve opening, *n* (%)	23 (38)
Aortic regurgitation, *n* (%)	10 (16)
Mitral regurgitation, *n* (%)	3 (5)
Tricuspid regurgitation, *n* (%)	11 (20)
Estimated right atrial pressure, *n* (%)	
3 mmHg	46 (77)
8 mmHg	8 (13)
15 mmHg	6 (10)
Post-operative period (days)	73 [25–936]
TTE–CT interval (days)	2 [−1 to 18]

Ejection fraction was measured using the modified Simpson’s method when feasible; otherwise, the Teichholz method was used. Aortic valve opening was considered present if opening was observed in at least 1 out of 10 cardiac cycles. The number of cases with aortic regurgitation, mitral regurgitation, and tricuspid regurgitation includes those with mild to moderate or greater severity.

### Diagnosis of OG structural abnormalities using TTE

Among the 62 examinations, 18 (12 in the proximal portion, 7 in the distal portion, and 1 in both portions) were diagnosed with OG structural abnormalities on the CT examinations performed simultaneously. Of these, 10 cases had bending and 9 had stenosis. In all patients, the OG was visualized to an extent sufficient for TTE-based evaluation. The sensitivity and specificity of TTE for detecting the abnormalities was 61% [95% CI 36–83%] and 100% [95% CI 92–100%], respectively. When OG structural abnormalities were evaluated separately for the proximal and distal portions, the sensitivity and specificity were 33% [95% CI 10–65%] and 100% [95% CI 93–100%] for the proximal portion, and 71% [95% CI 29–96%] and 100% [95% CI 94–100%] for the distal portion, respectively. When OG structural abnormalities were evaluated separately for the types of abnormalities (bending or stenosis), the sensitivity and specificity were 50% [95% CI 19–81%] and 96% [95% CI 87–100%] for bending, and 56% [95% CI 21–86%], and 98% [95% CI 90–100%] for stenosis, respectively (*Table 2*).

**Table 2:** Diagnostic accuracy of TTE for detecting OG structural abnormalities

	Sensitivity	Specificity
Overall	61% [36–83]	100% [92–100]
Proximal portion	33% [10–65]	100% [93–100]
Distal portion	71% [29–96]	100% [94–100]
Bending	50% [19–81]	96% [87–100]
Stenosis	56% [21–86]	98% [90–100]

Data represents diagnostic performance measures with 95% CIs.

After excluding 8 overlapping cases from the total of 62 exams, diagnostic performance was evaluated in the remaining 54 cases. The sensitivity and specificity was 61% and 100% for overall OG abnormalities, respectively. When analysed by location, the sensitivity and specificity was 33% and 100% for the proximal portion, and 71% and 100% for the distal portion, respectively. When analysed by abnormality type, the sensitivity and specificity was 50% and 96% for bending, and 56% and 98% for stenosis, respectively.

In all seven cases with stenosis, the peak velocity measured by continuous-wave Doppler was ≥2.0 m/s.

In this study, only one case was clinically actionable with associated symptoms and device alarms, and subsequent surgical correction of the OG was performed.

## Discussion

The present study demonstrated that using our institutional protocol TTE can be a novel and accurate tool for detecting OG structural abnormalities. Although there have been case reports suggesting the usefulness of TTE for detecting OG structural abnormalities such as eOGO,^7^ this is the first study investigating the diagnostic accuracy of TTE.

Severe stenosis or occlusion of OG occurs in ∼6–7% of LVAD patients and includes conditions such as eOGO, anastomotic stenosis, and torsion.^1^ Associated symptoms and adverse events include heart failure, low pump flow, and pump alarms. The incidence of eOGO increases with longer durations of LVAD support.^2–5^

Regarding the incidence of OG structural abnormalities, the frequency observed in our cohort was higher than that reported in a previous multicentre study,^5^ with an incidence of 29% (18/62 cases). Several factors may explain this discrepancy. First, the reported incidence of 29% was derived from a subgroup analysis restricted to patients who underwent both CT and TTE. This subgroup may have selectively included patients in whom OG obstruction was clinically suspected and therefore reflects the frequency in a selected population from a single-centre, retrospective study rather than the true incidence in general clinical practice. Second, in contrast to the previous study,^5^ our analysis included both graft stenosis and bending. When the analysis was limited to stenosis alone, the incidence decreased to 15% (9/62 cases) in the subgroup with both CT and TTE data. Furthermore, the reported incidence of eOGO varies considerably across the literature, with rates ranging from ∼14% to 33% in other studies.^2,4^ Differences in study design, patient selection, and definitions of stenosis may contribute to this variability, making direct comparisons across studies difficult.

Although ∼50% stenosis does not typically cause flow reduction or symptoms, regular follow-up is recommended due to the potential for progression.^5,8^ Several reports have described methods for visualizing the OG; however, the diagnostic accuracy of TTE for OG structural abnormalities has remained unclear. In this study, using our standardized institutional protocol, we demonstrated that TTE is a non-invasive and useful tool for detecting OG structural abnormalities in patients with LVAD, with particularly high diagnostic accuracy for the distal portion of the OG. The subcostal approach allowed for evaluation of the proximal portion of the OG without interference from artefacts caused by bones or lungs; however, several factors may have limited the extent of OG visualization in this region, including the proximity of the driveline and tenderness in the subcostal area. The use of colour Doppler imaging for proximal OG assessment was sometimes poor by reverberation artefacts originating from the pump. These factors may have contributed to the inability to detect structural abnormalities in certain patients. A CW Doppler peak velocity ≥2.0 m/s may serve as a useful supportive screening threshold for OG stenosis. However, flow velocity is influenced by device-specific graft diameter, pump settings, and Doppler angle alignment; therefore, a single universal cut-off value cannot be proposed for definitive diagnosis, and Doppler findings should be interpreted in conjunction with morphological assessment.

Although TTE demonstrated high specificity, its limited sensitivity indicates that contrast-enhanced CT remains indispensable for the diagnosis of OG structural abnormalities. However, TTE can be useful and repeatable as a complementary and non-invasive tool for screening and follow-up. Further improvement of visualizing protocol and its sensitivity are expected.

Several limitations should be acknowledged in the present study. First, we acknowledge that the inclusion of multiple LVAD models represents an important limitation of this study. Differences in LVAD model and flow characteristics may influence the flow patterns assessed by TTE. However, due to the limited sample size, device-specific subgroup analyses were not feasible. Although various LVAD devices were included, the course of the outflow graft is fundamentally similar across devices, and we consider that there were no notable device-specific differences in OG visualization or diagnostic performance. Second, as this was a retrospective study, data on interobserver and intraobserver reproducibility were not available.

## Conclusions

Although it has relatively low sensitivity compared to CT, TTE can be a non-invasive and repeatable tool for detecting OG structural abnormalities in patients with LVAD. Further optimization is required to improve the accuracy of TTE, especially in evaluating the proximal portion.

## Data Availability

The data underlying this study may be available from the corresponding author upon reasonable request.

## Lead author biography

Tasuku Sato, PhD, works as a Clinical Laboratory Technologist at the Heart Center, Kyushu University Hospital, Fukuoka, Japan. Clinical responsibilities include a wide range of cardiovascular ultrasound examinations, such as imaging of left ventricular assist devices, congenital heart disease, valvular heart disease, ischaemic heart disease, 3D transthoracic echocardiography, and vascular ultrasound. Additional duties include intraprocedural support in the cardiac catheterization laboratory. Research activities focus on cardiovascular imaging, with seven peer-reviewed publications in this field.
